# *TMEM45A* Is Dispensable for Epidermal Morphogenesis, Keratinization and Barrier Formation

**DOI:** 10.1371/journal.pone.0147069

**Published:** 2016-01-19

**Authors:** Aurélie Hayez, Edith Roegiers, Jérémy Malaisse, Benoit Balau, Christiane Sterpin, Younes Achouri, Catherine Lambert De Rouvroit, Yves Poumay, Carine Michiels, Olivier De Backer

**Affiliations:** 1 URPhyM-NARILIS, University of Namur, Namur, Belgium; 2 URBC-NARILIS, University of Namur, Namur, Belgium; 3 Université Catholique de Louvain, de Duve Institute, Brussels, Belgium; Columbia University Medical Center, UNITED STATES

## Abstract

*TMEM45A* gene encodes an initially uncharacterized predicted transmembrane protein. We previously showed that this gene is highly expressed in keratinocytes where its expression correlates with keratinization, suggesting a role in normal epidermal physiology. To test this hypothesis, we generated *TMEM45A* knockout mice and found that these mice develop without any evident phenotype. The morphology of the epidermis assessed by histology and by labelling differentiation markers in immunofluorescence was not altered. Toluidine blue permeability assay showed that the epidermal barrier develops normally during embryonic development. We also showed that depletion of *TMEM45A* in human keratinocytes does not alter their potential to form *in vitro* 3D-reconstructed epidermis. Indeed, epidermis with normal morphogenesis were generated from *TMEM45A*-silenced keratinocytes. Their expression of differentiation markers quantified by RT-qPCR and evidenced by immunofluorescence labelling as well as their barrier function estimated by Lucifer yellow permeability were similar to the control epidermis. In summary, *TMEM45A* gene expression is dispensable for epidermal morphogenesis, keratinization and barrier formation. If this protein plays a role in the epidermis, its experimental depletion can possibly be compensated by other proteins in the two experimental models analyzed in this study.

## Introduction

The main function of the epidermis is to maintain an efficient barrier between the organism and its external environment. Keratinocyte is the main cell type in the epidermis. These cells undergo proliferation and terminal differentiation, producing the cornified layer, the outermost skin barrier. This layer is composed of dead keratinocytes in which the intracellular content has been replaced by a compact keratin network, and the plasma membrane internally reinforced by a rigid proteolipidic cornified envelope. The intercellular spaces are filled by a mostly lipidic “mortar” that participates to the impermeability of the barrier [1–3]. The complex process responsible for epidermal barrier maintenance is called keratinization.

Although morphological modifications of keratinocytes during keratinization have been well characterized, the precise molecular and cellular functions of many actors of this process remain largely unknown. We showed recently that expression of *TMEM45A* (also known as *DERP7*, *DNAPTP4* and *FLJ10134*) is strongly induced by differentiation in cultured human keratinocytes and correlates with keratinization in the granular layer of the epidermis. We also observed the same correlation in thymic keratinized epithelial cells inside Hassal bodies [4]. High-throughput RNA expression analyses similarly associated *TMEM45A* expression with skin and keratinization in human and mouse [5–8]. In addition, *Tmem45a* was identified as a candidate for positive regulation of embryonic epidermal growth [9]. Altogether, these observations suggested a role for *TMEM45A* in epidermal development and maintenance. However, the nature of this possible role remained completely unknown.

*TMEM45A* belongs to the large group of *TMEM* genes encoding uncharacterized proteins predicted to contain transmembrane (TM) domains. Human *TMEM45A* and mouse *Tmem45a* orthologs encode proteins sharing 64% of identical amino acids. We observed that TMEM45A protein is mainly located in the trans-golgi network (TGN) in cultured human keratinocytes and in the granular layer of epidermis [4]. No TMEM45A was observed in lysosomes or in corneodesmosin-containing lamellar bodies [4]. One important function of granular keratinocytes is secretion of lamellar bodies (LB) content, including precursors of the intercorneocyte matrix and their processing enzymes, enzymes involved in desquamation (and their inhibitors), antimicrobial peptides, and corneodesmosin, a protein that reinforces desmosomes [10]. The granular keratinocyte is thus a specialized secretory cell, whose LB are the only clearly identified secretion vesicles [10]. Endomembrane system and the associated secretion vesicles present in granular keratinocytes are complex and only partially characterized. Apical membrane of these cells exhibits many deep invaginations connecting together intercellular space, lamellar bodies and trans-golgi network [11]. Lipids are hypothesized to be transported into the Golgi apparatus and LB by membrane transporters, and then processed inside the Golgi apparatus [12, 13]. TGN and LB are proposed to be heterogeneous compartments in granular keratinocytes, as some cargoes are independently synthetized and transported as separated aggregates [14, 15].

In order to unveil a role for TMEM45A in epidermis, we generated KO mice. To the best of our knowledge, this is the first time that such KO mice are reported. Furthermore, we invalidated TMEM45A expression in 3D-reconstructed human epidermis and in autocrine monolayer cultures of human keratinocytes.

## Materials and Methods

### Antibodies and chemicals

Antibodies and chemicals are described in supplementary data (S1 File).

### Generation of *Tmem45a* knockout mice

**Fig 1:**
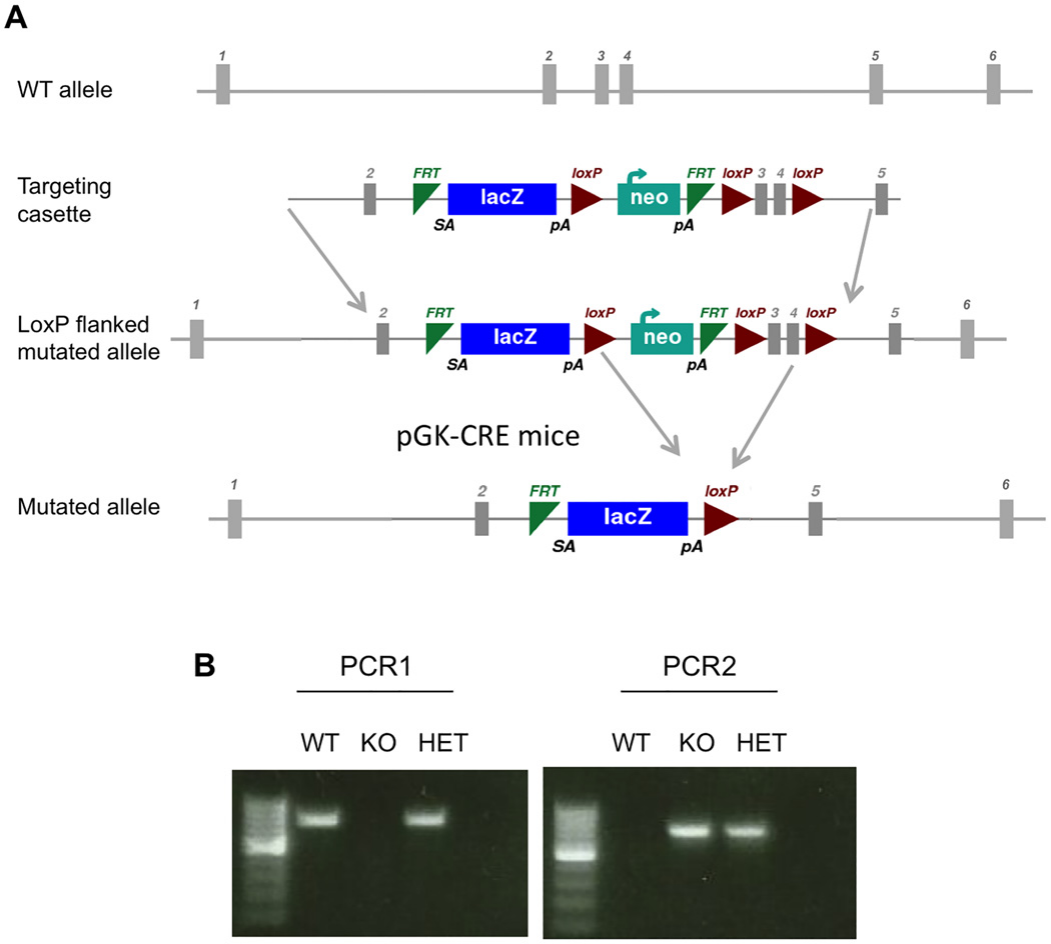
Gene targeting of *Tmem45a* by homologous recombination in embryonic stem cells. **(A)** Schematic representation of the WT *Tmem45a* allele, of the targeting vector, of the knockout first allele and allele obtained after Cre-mediated recombination. **(B)** *Tmem45a* knockout mice genotyping. PCR1 amplified the WT allele and PCR2 is specific of the targeted allele.

The *Tmem45a* targeting vector PG00253_Z_5_A04 was obtained from the International Mouse Phenotyping Consortium (IMPC). Homologous recombination of this vector in ES cells produced a conditional “knockout first” allele with a PGK-neomycin cassette and a LacZ reporter gene are inserted between the second and the third *Tmem45a* exons (*Tmem45a*^*tm1a(KOMP)Mbp*^ allele, Fig 1A). The targeting vector was introduced by electroporation in 129/Ola embryonic stem cells (ES14Tg2a). Gene replacement was detected by PCR (Primer-Forward: 5’-AGATGGCGCAACGCAATTAAT-3’; Primer-Reverse: 5’-GGAATAACCCAGAGGTCCAT-3’). Integrity of the recombined *Tmem45a* allele was verified by Southern blot analyses (B*am*HI digestion) using probes corresponding to nucleotides 56764 to 57475 (NC_000082.6) and to LacZ sequences (data not shown). Embryonic stem cells containing the recombined allele were injected into C57BL/6 blastocysts and reimplanted into CD1 pseudopregnant females. One chimeric male bearing the recombined allele was mated with C57BL/6 female mice to obtain heterozygotes *Tmem45a*^*tm1a(KOMP)Mbp*^.

These mice were mated with PGK-Cre transgenic mice to obtain animals with allele *Tmem45a*^*tm1b(KOMP)Mbp*^, with deleted PGK-Neo and *Tmem45a* exons 3 and 4 (Fig 1A). Homozygous *Tmem45a*^*tm1b(KOMP)Mbp*^ males and females were obtained by subsequent crossings.

All mice used in this work have a mixed 129/Ola and C57BL/6 genetic background. The approval of the local Ethic committee of the University of Namur was obtained for all the experiments, which were performed according to the European legislation.

### Mouse genotyping

Genotyping of animals was performed by PCR using DNA extracted from tails. The WT Tmem45a allele was amplified by PCR1 (PCR1_F primer: GTTCTACAACCACACACACG and PCR1_R primer: CTGAGTTATTCTAGGCAGGG) and gives a 758 bp fragment. *Tmem45a*^*tm1b(KOMP)Mbp*^ allele was amplified by PCR2 (using PCR2_F primer: TCACCCGAGTGTGATCATCT and PCR2_R primer: GGTAGTTCAGGCAGTTCAA). Amplified fragment length from the HET or KO mouse is 691 bp (Fig 1B).

### Isolation of normal human epidermal keratinocytes

Normal human adult abdominal skin was obtained from plastic surgery of healthy subjects who had given their informed consent. They were processed to isolate primary keratinocytes, as described in [16].

### Lentiviral particles

MISSION pLKO.1-puro vector—based lentiviral particles containing a shRNA cassette under the U6 promoter and a puromycin resistance gene were produced by Sigma-Aldrich (St Louis, MO, US). The shRNA targeting *TMEM45A* exon 4 (TMEM45A shRNA; sequence 5’-GAGTTCCTTGTTCGGAACAAT-3’; nucleotide position 821–841 on the mRNA sequence (NCBI Reference: NM_018004.1)) was chosen. As negative control, a non-target shRNA that does not target any known mammalian gene (NT shRNA; 5’-CCGGCAACAAGATGAAGAGCACCAACTC-3’) was used.

### Human keratinocyte transduction

Secondary cultures of keratinocytes were trypsinized, as described in [16] and the pellet was resuspended in growth medium containing 4 μg/ml protamine sulfate to increase infection efficiency. Lentiviral particles containing non-target shRNA (NT shRNA) or shRNA targeting *TMEM45A* (TMEM45A shRNA) were added to the cell suspension at a multiplicity of infection (MOI) of 10. Cells were seeded at a density of 7,000 cells/cm^2^. 24 hours post-infection, transduced cells were selected in medium containing 2 μg/ml puromycin.

### Reconstruction of human epidermis from transduced keratinocytes

When keratinocytes cultures transduced with NT or TMEM45A shRNA covered 80% of the flask area, cells were trypsinized and used to generate reconstructed human epidermis on polycarbonate filter, as described in [17], except that 250,000 transduced keratinocytes were seeded per insert. Reconstructed epidermis were processed for histology, as described in [17].

### Confluence-induced differentiation of autocrine transduced monolayers of human keratinocytes

When keratinocyte cultures reached 50–60% of cell coverage on the substratum, medium was replaced by fresh medium for autocrine growth. Confluent cultures stop to proliferate and begin to differentiate, as shown by the expression of differentiation markers [16].

### RT-qPCR

Total RNA was isolated using High Pure RNA isolation kit (Roche, Basel, Switzerland) for monolayers and RNeasy mini kit (Qiagen, Hilden, Germany) for reconstructed human epidermis and mouse tissues, according to manufacturer’s instructions. 1 μg of total RNA was reverse transcribed using SuperScript II kit (Life Technologies, Carlsbad, CA), according to the manufacturer’s instructions. PCR assays contained 300 nM of primers and FastStart Universal SYBR Green Master (Rox) (Roche, Basel, Switzerland). mRNA expression levels were quantified using the threshold cycle method on a 7300 real-time PCR system from ABI (Life Technologies, Carlsbad, CA) and normalized to the geometric mean of *RPLP0* and *TBP* reference genes values [16]. Primer sequences for human and mouse genes and for the puromycin resistance gene are described in supplementary data (S1 File).

Independent experiments were performed in triplicates. Values were expressed as relative quantification level with error bars representing 95% confidence intervals. Data were analyzed by paired *t*-test or two-way analysis of variance.

### Immunofluorescence detection in keratinocytes, reconstructed human epidermis and tissues

Immunofluorescence detection is described in supplementary data (S1 File).

### Transmission Electron Microscopy

Punches of 3 mm diameter from reconstructed human epidermis were fixed in 2.5% glutaraldehyde-formaldehyde buffer (0.1 M sodium cacodylate pH 7.4) overnight at 4°C and then washed with 0.2 M Sorensen phosphate buffer. Samples were post-fixed with 1% OsO_4_ in buffer containing 0.05 M phosphate buffer (Sorensen buffer dilution 1:4) and 0.25 M glucose for one hour at room temperature. Punches were dehydrated in ascending ethanol series up to ethanol 100% and incubated with propylene oxide. They were then progressively embedded in ascending propylene oxide/epoxy resin series (Araldite 502/Embed 812). After polymerization, ultrathin sections were mounted on grids and post-stained with uranyl acetate and lead citrate. Tissues were observed and pictured under a TECNAI 10 transmission electron microscope (FEI, OR).

### Western blot analysis of mouse tissues

Protein extraction from specific organs was performed after immerging organs in lysis buffer (20 mM MES, 30 mM Tris, 100 mM NaCl, 1% Triton X100, 20 mM NEM) containing a protease inhibitor mixture (cOmplete from Roche Molecular Biochemicals, dilution 1:25) and phosphatase inhibitors (1 mM NaVO_3_, 10 mM p-nitrophenyl phosphate, 10 mM β-glycerophosphate and 5 mM NaF) on ice. After centrifugation for 10 min at 13,000 rpm and 4°C, a Pierce protein quantification was performed. 5x loading buffer (0.5 M Tris-HCl pH 6.8, 20% SDS; 2-β-mercapoethanol, 30% glycerol, bromophenol blue) was added to the samples. Then samples were heated at 37°C for 3 min before analysis by electrophoresis performed on 10% polyacrylamide gel (Bio-Rad, Hercules, CA, US). Proteins were transferred onto PVDF membranes, blocked for 1 hour at room temperature with blocking solution (LI-COR, Lincoln, NE). Anti-TMEM45A was used as primary antibody and incubated O/N at 4°C. After washing in PBS- 0.1% Tween, the membrane was incubated with anti-rabbit IRDye-labelled antibody for 1 hour at RT. After washing, the membrane was scanned using an Odyssey infrared imaging system (LI-COR, Lincoln, NE). β-actin was used as loading control.

### Western blot analysis of human cultured keratinocyte monolayers

Human keratinocyte cultured as monolayers were lyzed with 0.05 M Hepes pH 7.4, 0.15 M NaCl, 0.1% SDS, 1% NP40, 1% Triton 1%, PIC (cOmplete from Roche Molecular Biochemicals, dilution 1:25) and PIB (1 mM NaVO_3_, 10 mM p-nitrophenyl phosphate, 10 mM β-glycerophosphate and 5 mM NaF). After Pierce protein quantification, WB was performed according to standard protocols, except that samples were not boiled before loading, but incubated 3 minutes à 37°C. Indeed, like TMEM45B [18], TMEM45A shows thermal aggregation in SDS—PAGE gels when samples are heated at 100°C.

### *In situ* staining of β-galactosidase activity in mouse tissues

Tail skin and other tissues were obtained from euthanized mice. Tissues were rinsed in PBS and fixed 30 min on ice in a solution containing 0.2% glutaraldehyde, 5 mM EGTA and 2 mM MgCl_2_ diluted in 0.1 M phosphate buffer pH 7.3. Tissues were washed 3 times during 15 min in washing buffer containing 0.02% NP-40, 0.01% sodium deoxycholate and 2 mM MgCl_2_ diluted in 0.1 M phosphate buffer pH 7.3. Tissue samples were stained in a solution containing 0.02% NP-40, 0.01% sodium deoxycholate, 2 mM MgCl_2_, 5 mM potassium ferricyanide diluted in 0.1 M phosphate buffer pH 7.3 with 1 mg/ml of X-gal diluted in N,N-dimethylformamide during 16 hours. Then, tissues were washed with PBS and fixed during 8 hours with 4% PFA. They were embedded in paraffin, and processed for histology. After coloration with Hemalun Erythrosin Safran, tissues were observed under an Olympus AX70 microscope (Tokyo, Japan).

### Toluidine blue exclusion

*Tmem45a*^*+/-*^ females were bred with *Tmem45a*^*+/-*^ males. 16.5 days post-coïtum, females were euthanized and embryos collected. Embryos or neonatal mice were euthanized on ice. Tails were kept for genotyping. Embryos were washed successfully in increased methanol baths (25%, 50%, 75% and finally 100%) and decreased methanol baths during 1 min each before staining for 30 min in 0.1% of toluidine blue diluted in PBS. Then, embryos were washed twice with PBS and fixed in 4% formalin-acetic acid solution.

### Permeability of reconstructed epidermis to Lucifer Yellow

Inserts were incubated in a 24 wells-plate containing 200 μl of growth medium per well. A 1 mM Lucifer Yellow solution was prepared in culture growth medium. After filtration, 150 μl of the Lucifer Yellow solution was added on top of each culture. After 6 hours of incubation in darkness, reconstructed epidermis were washed three times with PBS, fixed and processed as described [17]. Fluorescence of LY was observed under an AX70 Olympus microscope (Tokyo, Japan). Quantification of the Lucifer Yellow fluorescence contained in medium under the tissue was performed using Fluoriscan ASCENT spectrofluorimeter (Thermo scientific, MA) (excitation 485 nm; emission 535 nm).

### pH measurement

pH at the surface of the cornified layer was measured using a skin dedicated pH meter (Skin-pH-Meter PH 905, Courage+Khazaka Electronic GmbH, Köln, Germany).

## Results

### Invalidation of *TMEM45A* in mouse and human keratinocytes does not disturb epidermal development and maintenance

To determine whether *Tmem45a* is required for epidermal development or homeostasis, we generated for the first time *Tmem45a* knockout mice. Mice harboring a “knockout first” conditional (floxed) LacZ-tagged Tmem45a allele (*Tmem45a*^*tm1a(KOMP)Mbp*^) were generated using a targeting vector developed by the International Mouse Phenotyping Consortium (IMPC) (Fig 1). In this allele, the splice acceptor of the LacZ cassette captures the RNA transcript and an efficient polyadenylation signal truncates the transcripts so that the part of the gene downstream from the cassette (exons 3 to 5) is not transcribed into mRNA. This allele only has the potential to encode a peptide corresponding to the first 63 aa of Tmem45A encoded by exon 2 (exon 1 is not coding). The LacZ present in this allele reports transcriptional activity of the TMEM45A promoter. As exons 3 and 4 are flanked by LoxP sites, crossing with PGK-Cre deleter mice allowed us to generate mice with deleted exons 3 and 4 (*Tmem45a*^*tm1b(KOMP)Mbp*^ allele, noted *Tmem45a*^-^ *hereafter*). This allele is a null allele because the deletion results in a frameshift in exon 5 that should result in non-sense mediated decay.

*Tmem45a*^*+/-*^ heterozygous mice were viable and fertile. Interbreeding of these mice produced pups with normal mendelian ratio, including 25% of homozygous *Tmem45a*^*-/-*^ mice. Gross morphological and histopathological features of these knockout mice were normal, including the epidermis (data not shown). Weight gain was not different from wild-type littermates.

**Fig 2:**
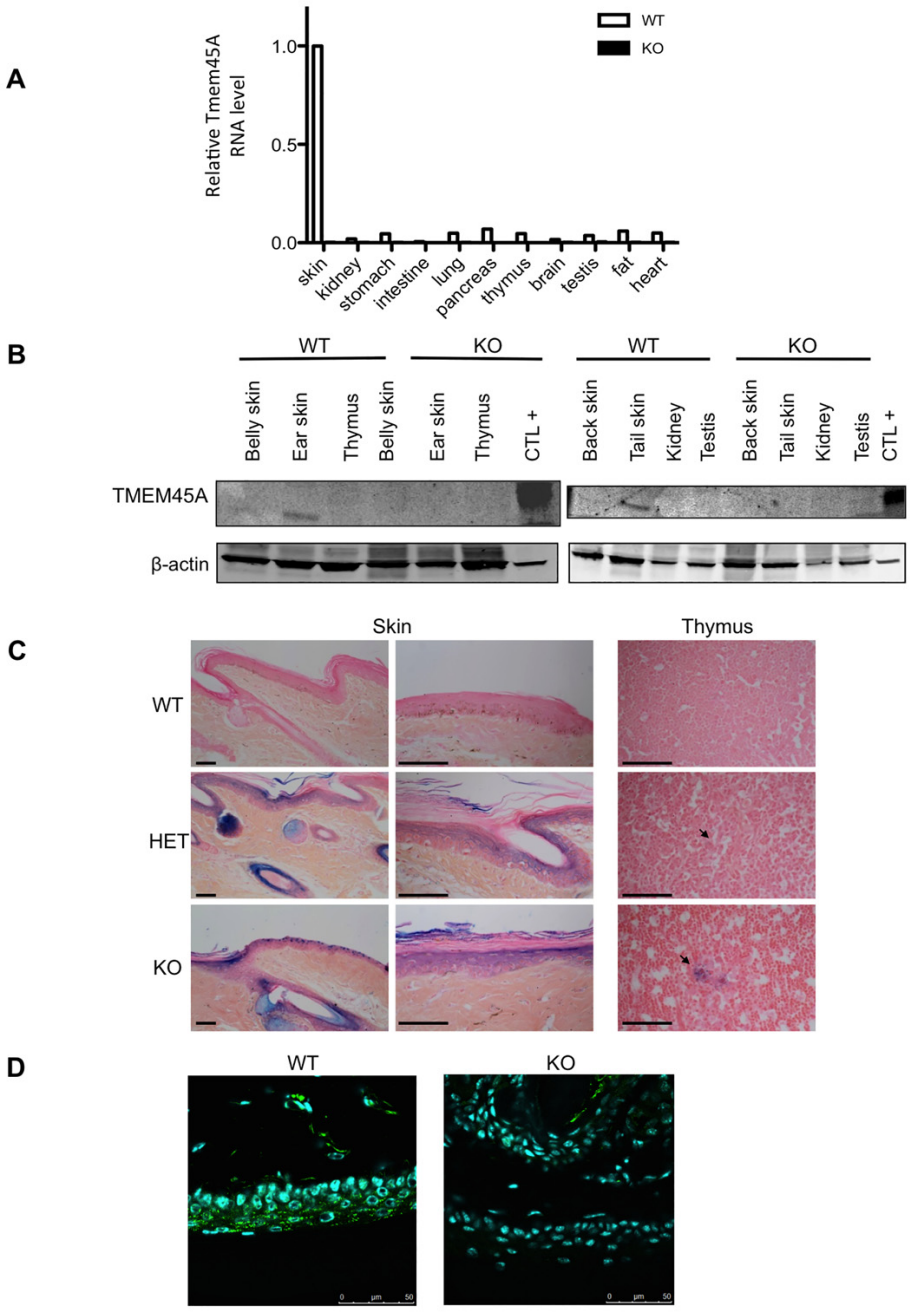
Absence of expression of *Tmem45a* in the *Tmem45a*^-/-^ mouse. **(A)** Quantification by RT-qPCR of *Tmem45a* mRNA level in different organs from wild type (WT) and knockout (KO) mice. The RNA levels are represented in arbitrary units, with a value of 1.00 corresponding to the level in WT skin. (**B)** Western blot analysis of TMEM45A abundance in tissues from wild type (WT) and Tmem45a knockout (KO) adult mice. β-actin was used as the loading control. Different organs and different skin anatomical location were analyzed in the two western blots. “CTL+” corresponds to a protein extract of HEK293 cells transfected with a plasmid encoding the human TMEM45A cDNA. (**C)** X-gal staining of the LacZ reporter activity in tail skin and thymus tissues of WT, Tmem45^+/-^ (HET) and Tmem45^-/-^ (KO) mice. Tissues were also stained with Hemalun Erythrosin (HE). Arrows indicate Hassal bodies. Scale bars: 50 μm. **(D)** Immunofluorescence detection of TMEM45A in tail skin of WT and KO mice. Scale bars: 50 μm. (B, C, D). The presented data are representative of the results obtained for at least three independent experiments.

RT-qPCR proved that the residual level of *Tmem45a* mRNA was reduced to background in all tested organs (Fig 2A) as well as in several anatomical location of skin (ear, tail, belly and back, data not shown), indicating that, in case a truncated TMEM45A protein is produced, its level could only be very low. The possible expression of a truncated protein could not be investigated because the only available TMEM45A antibody recognizes a peptide located in the C-terminal part of the protein, which is missing in the knockout animals. The 31 kDa band detected by Western blot analysis of TMEM45A in skin of wild type mouse was absent in *Tmem45a*^*-/-*^ skin (Fig 2B), confirming the absence of *Tmem45a* in the KO mice.

We used the LacZ reporter to investigate the activity of the *Tmem45a* promoter (and thus the putative expression of *Tmem45a* mRNA) in mouse tissues by *in situ* X-gal staining of β-galactosidase activity (Fig 2C). X-gal staining was observed in the suprabasal epidermal layers, hair follicles, sebaceous glands and thymic Hassal bodies. Similar staining patterns were observed in *Tmem45a*^*tm1a(KOMP)Mbp*^ heterozygotes and homozygous mice. As expected, no LacZ staining was observed in wild type control mice.

A direct detection of the TMEM45A protein was performed by immunofluorescence labelling on skin sections from the tail (the same anatomical location than in Fig 2C). These results showed that TMEM45A is expressed in the suprabasal layers of the epidermis of WT mice but not in the epidermis of the KO mice (Fig 2D). Furthermore, the labelling was granular, which is consistent with the subcellular localization of the protein in the Golgi apparatus. Finally, we also isolated mouse embryonic fibroblasts (MEFs) from three different pairs of WT and KO littermates and the expression of TMEM45A protein was detected by immunofluorescence labelling. The protein was only detected in WT MEFs incubated under hypoxia. These results are consistent with the fact that TMEM45A is highly expressed in skin but not in other tissues (the protein was not detected in normoxic MEFs) but that its expression is induced by hypoxia [19]. Again, the fluorescence labelling was granular and localized on one side of the nucleus, which is consistent with a Golgi apparatus location (S1 Fig). On the other hand, no labelling was observed in KO MEFs, even under hypoxia conditions.

We used another experimental model to investigate the possible role of *TMEM45A* in keratinocyte differentiation: reconstructed human epidermis (RHE), a simplified epidermal tissue obtained *in vitro* after culture of human keratinocytes on polycarbonate filters [17].

**Fig 3:**
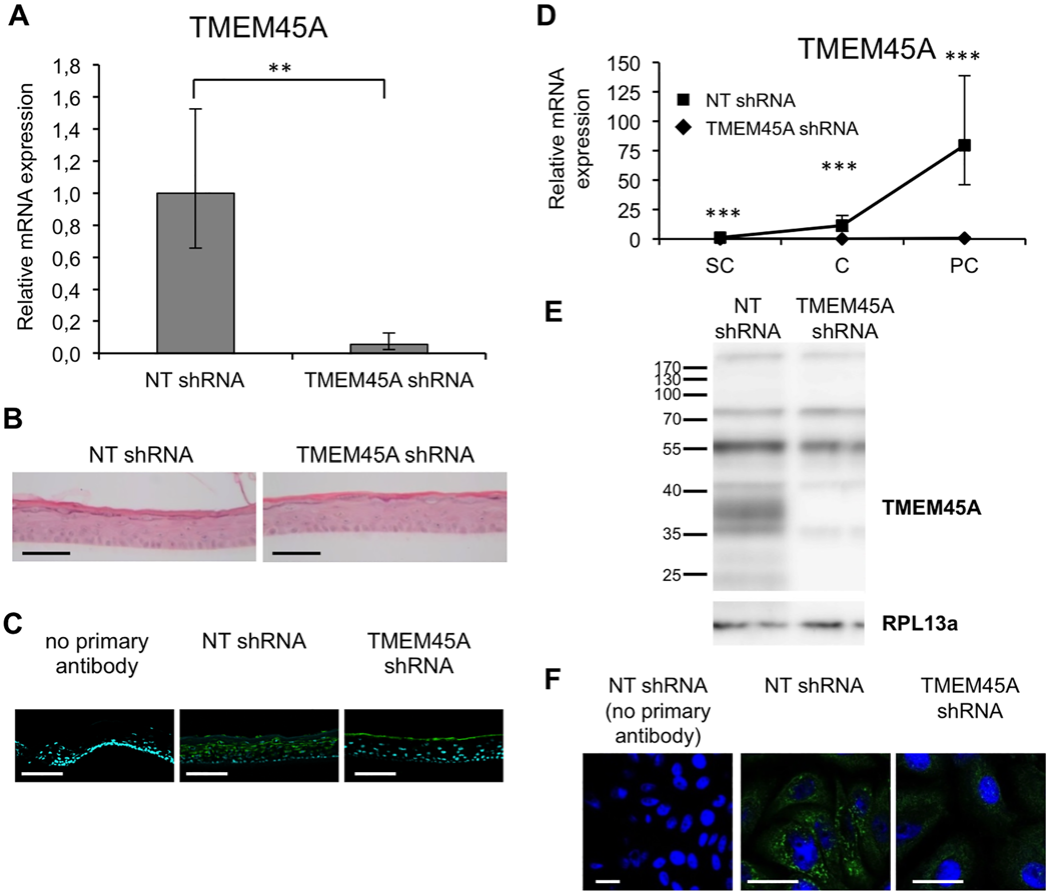
Efficient invalidation of *TMEM45A* in RHE after 11 days of reconstruction (A-C) and in monolayer culture of human keratinocytes (D-F). **(A)** Relative quantification of TMEM45A mRNA levels in epidermis reconstructed with keratinocytes transduced with NT shRNA or shRNA targeting TMEM45A. Bars represent 95% confidence intervals. Paired t-test (n = 3, **p≤0.1). **(B)** Morphology of HE-stained epidermis. Bars: 50 μm. **(C)** Immunofluorescence detection of TMEM45A in RHE. Bars: 50 μm. **(D)** Relative mRNA quantification in monolayers at subconfluence (SC), confluence (C) and post-confluence (PC). Bars represent 95% confidence intervals. ANOVA 2 (n = 3, *** p≤0.001). **(E)** TMEM45A abundance analysis by WB in post-confluent monolayer culture. RPL13a is the loading control. **(F)** Detection of TMEM45A in confluent monolayers culture. Bars: 25 μm. (B, C, E, F). The presented data are representative of the results obtained for at least three independent experiments.

**Fig 4:**
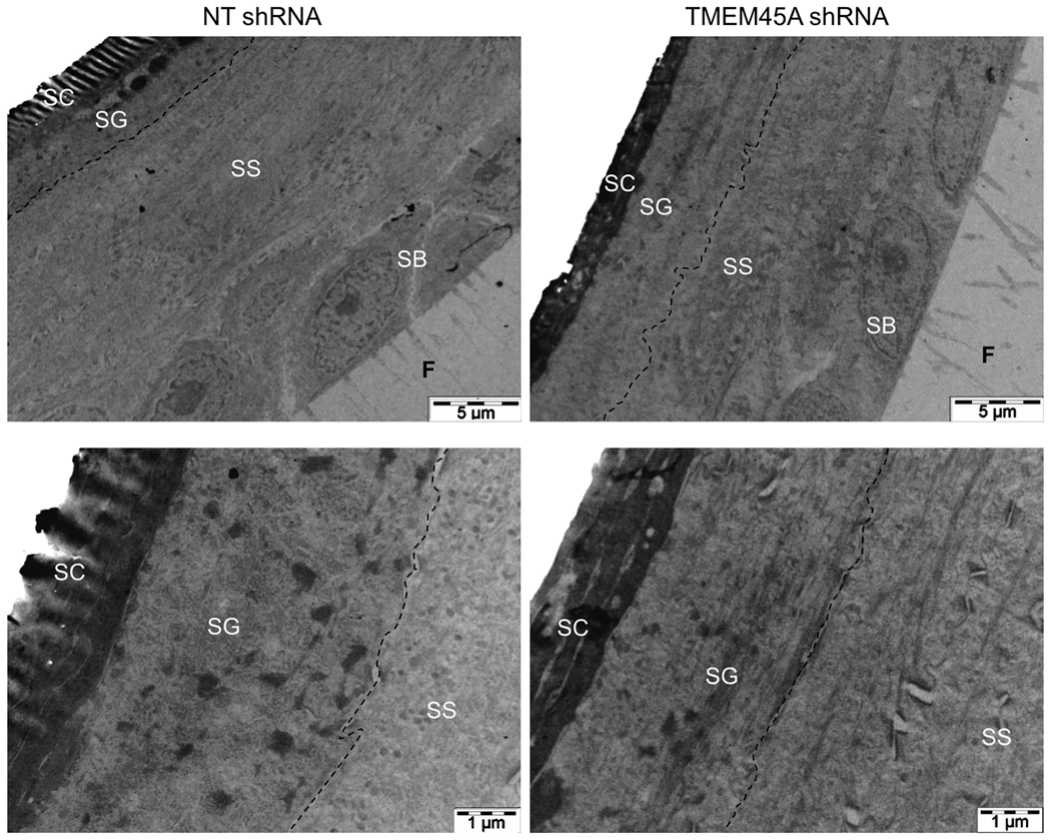
*TMEM45A*-silencing has no obvious impact on the morphology of human reconstructed epidermis in transmission electron microscopy. Reconstructed epidermis from keratinocytes transduced with NT or TMEM45A shRNA after 11 days of reconstruction were processed for transmission electron microscopy. SC: stratum corneum. SG: stratum granulosum. SS: stratum spinosum. SB: stratum basale. F: filter. The dotted lines delineate the border between SS and SG.

**Fig 5:**
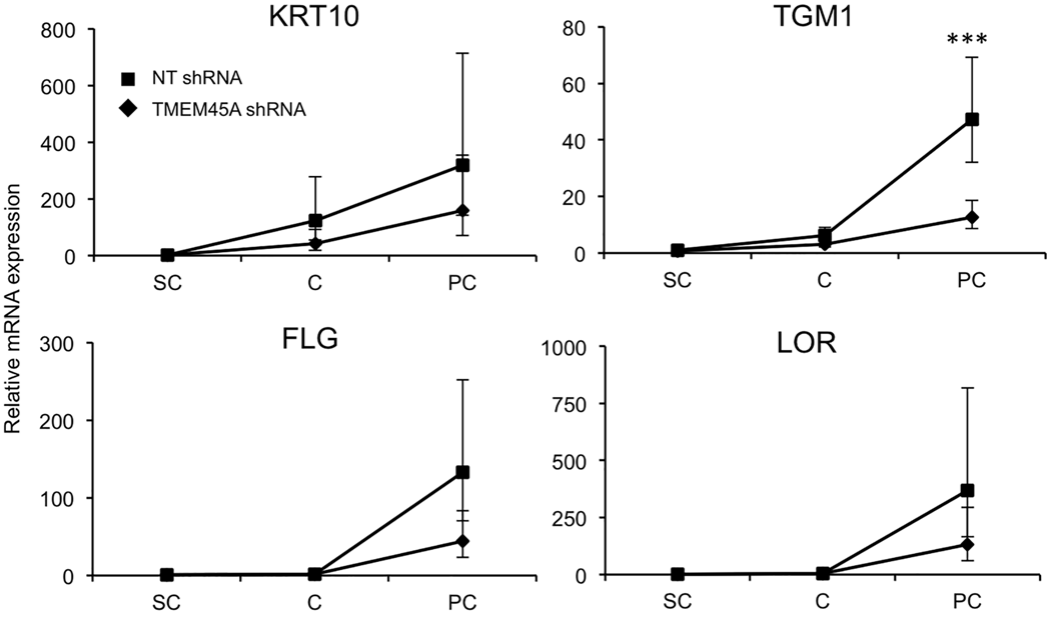
*TMEM45A* invalidation does not alter confluence-induced differentiation of keratinocytes. Relative quantification of KRT10, TGM1, FLG and LOR mRNA expression levels was performed after RNA extraction from human keratinocytes grown as autocrine monolayers after being transduced with NT or TMEM45A shRNA at subconfluence (SC), confluence (C) and post-confluence (PC) of the culture. Bars represent 95% confidence intervals. ANOVA 2 (n = 3, *** p≤0.001).

**Fig 6:**
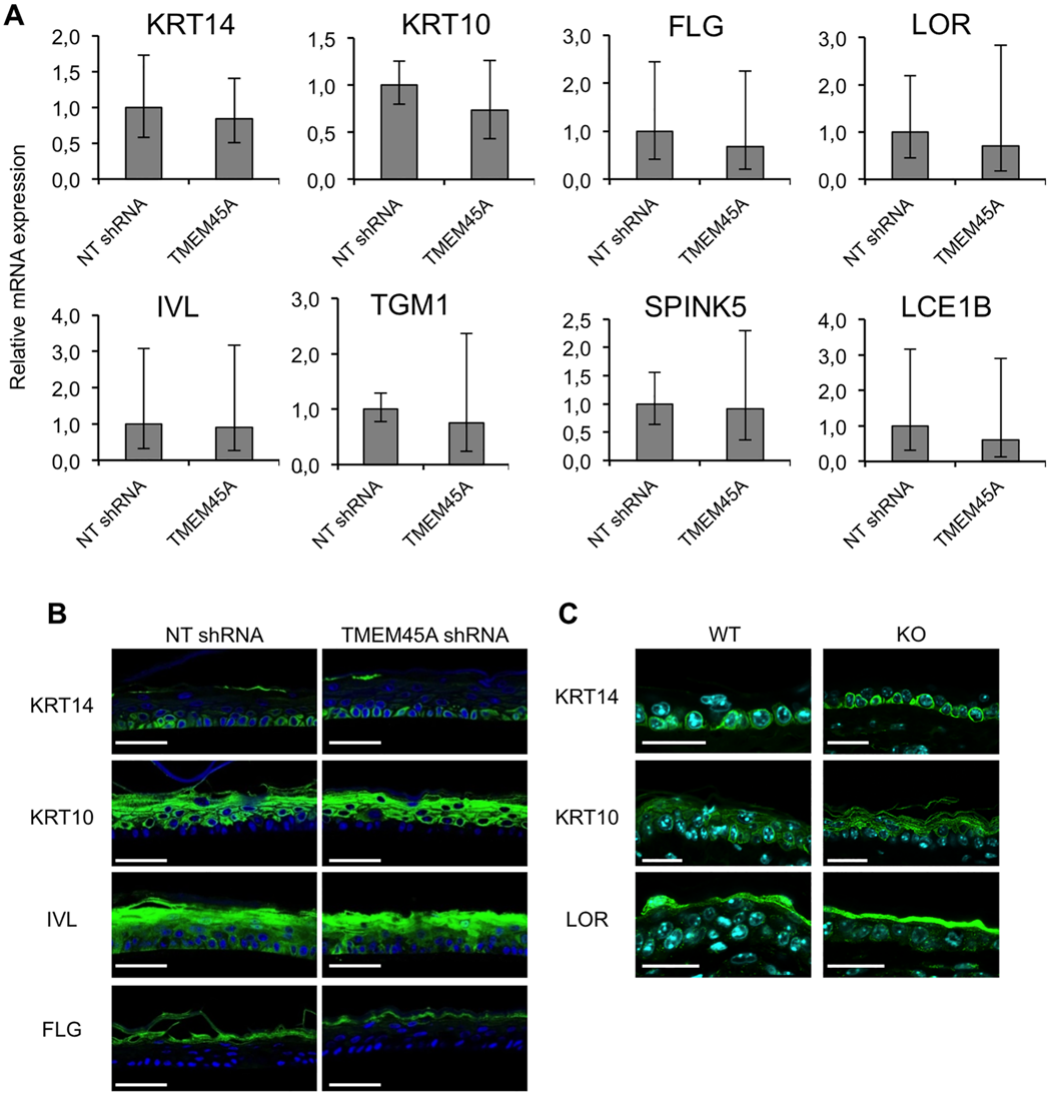
*TMEM45A* expression is not essential for keratinization. **(A)** Relative quantification of KRT14, KRT10, FLG, LOR, IVL, TGM1, SPINK5 and LCE1B mRNA levels in reconstructed epidermis obtained from human keratinocytes transduced with NT shRNA or TMEM45A shRNA after 11 days of reconstruction. Bars represent 95% confidence intervals. Paired t-test (n = 3). No statistically significant difference was observed. **(B)** Immunofluorescence detection of KRT14, KRT10, IVL and FLG in reconstructed epidermis after 11 days of reconstruction. Scale bars: 50 μm. **(C)** Immunofluorescence detection of KRT14, KRT10 and LOR in WT and KO mouse adult ear skin. Scale bars: 25 μm. (B, C) The presented data are representative of the results obtained for at least three independent experiments.

We invalidated *TMEM45A* in human epidermal keratinocytes by transduction of lentiviral particles designed to express shRNA targeting *TMEM45A*. RT-qPCR and Western blot analysis showed that *TMEM45A* mRNA and protein were actually depleted in transduced cells (Fig 3). We observed that *TMEM45A*-invalidated keratinocytes were able to generate RHE exhibiting normal morphology under optical and electron microscopy (Figs 3B and 4). TMEM45A-depleted keratinocytes were also cultured in autocrine monolayers and induced to differentiate by cell confluence (Fig 3D–3F). The mRNA relative expression of early (*KRT10*) and late (*TGM1*, *FLG* and *LOR*) markers of differentiation was assessed at sub-confluence, confluence and post-confluence stages. No significant alteration was observed, except for *TGM1*, but a decreased mRNA level for all markers was noticed in *TMEM45A*-deficient cultures (Fig 5). Keratinization of *TMEM45A*-invalidated RHE was also monitored by measuring expression of genes involved in differentiation (*KRT14*, *KRT10*, *FLG*, *LOR*, *IVL*, *TGM1*, *FLG*, *SPINK5* and *LCE1B*). The relative mRNA levels of these genes were found unaltered (Fig 6A). *In situ* detection by immunofluorescence labelling of keratin-14, keratin-10, involucrin and filaggrin further indicated that *TMEM45A* invalidation did not modify the histological localization of these proteins (Fig 6B). Similarly, no alteration of keratin 14, keratin 10 or loricrin was observed in the epidermis of *Tmem45a*^-/-^ mice (Fig 6C). Together, these observations indicate that *TMEM45A* is not required for epidermal differentiation and keratinization.

### The epidermal barrier function is not altered by *TMEM45A* deficiency

**Fig 7:**
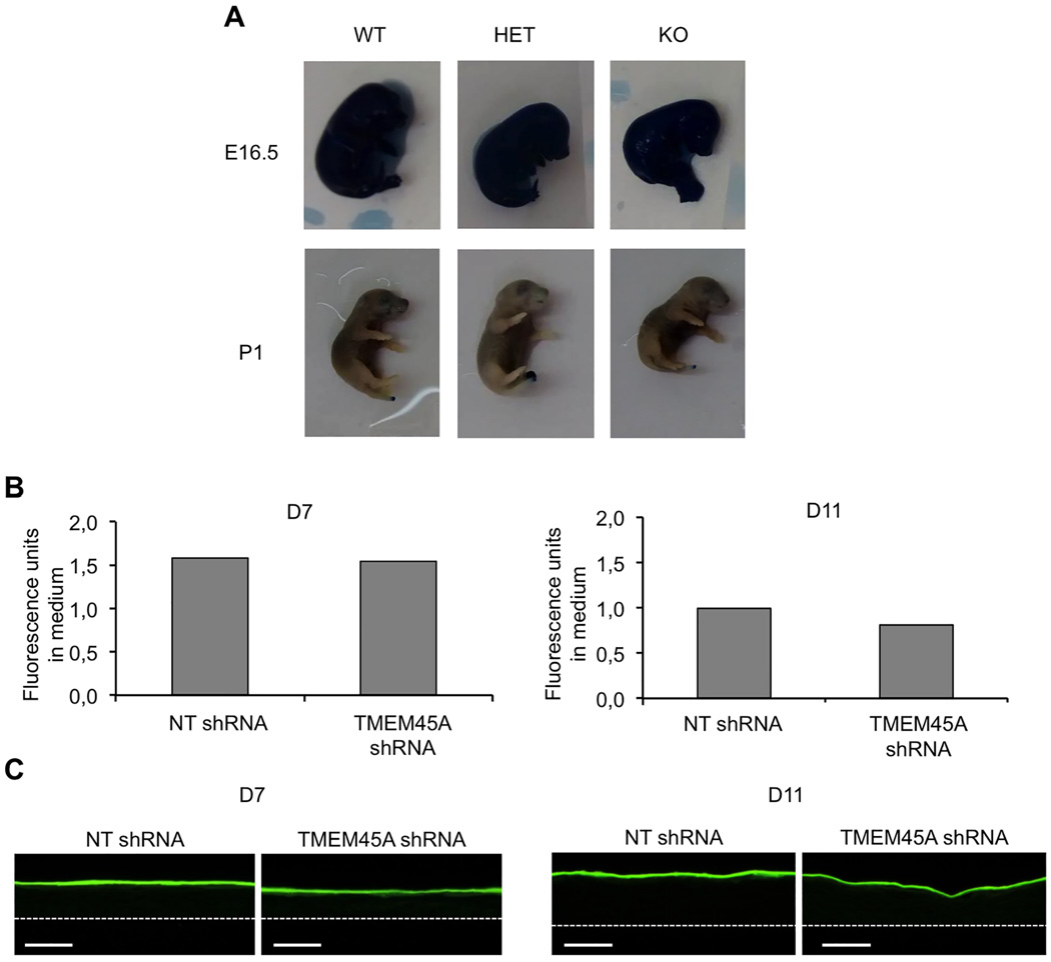
*TMEM45A* is not essential for the impermeability of the cornified layer. **(A)** Toluidine blue exclusion assay in E16.5 and P1 of WT, KO and HET mouse littermates. **(B-C)** Permeability of the cornified layer of human epidermis reconstructed from keratinocytes transduced with NT or TMEM45A shRNA to Lucifer Yellow, tested after 7 or 11 days of reconstruction (n = 1). **(B)** After the incubation, the fluorescence in medium was quantified. **(C)** Tissues were processed and analyzed using fluorescence microscopy. The dotted lines delineate the polycarbonate filter (scale bars: 50 μm).

Despite the normal expression of epidermal differentiation/keratinization markers, the epidermal barrier function could be disturbed in the absence of TMEM45A. We tested this function both in *Tmem45a* knockout mice and in TMEM45A-depleted RHE. *Tmem45a* knockout mice develop normally and are viable, excluding a severe barrier defect at birth or later. We investigated the establishing of barrier formation during development, using the toluidine blue whole-mount permeability assay [20]. No difference was observed between wild type, and knockout animals at E16.5 and P1, meaning before and after impermeability establishing (Fig 7A). At E17.5, we observed a high variability among embryos, which could not be related to the genotypes (S2 Fig). We concluded that skin impermeability is set up normally in the absence of *Tmem45a* during mouse development.

In RHE, a functional barrier also develops progressively [17]. We assessed the permeability of RHE at day 7 and 11 of reconstruction using the fluorescent Lucifer Yellow compound. Amount of Lucifer Yellow (LY) in the medium and histological analysis of RHE after Lucifer Yellow exposure showed no evidence of alteration caused by *TMEM45A* invalidation at both tested stages (Fig 7B and 7C). These results revealed the effective development of the barrier in *TMEM45A*-invalidated RHE.

**Fig 8:**
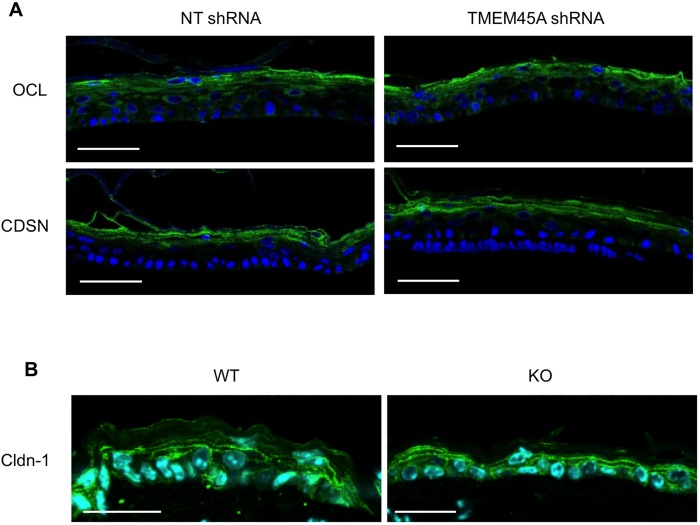
*TMEM45A* silencing does not alter tissue localization of junctional proteins. **(A)** Immunofluorescence detection of occludin (OCL) and corneodesmosin (CDSN) in RHE obtained after 11 days of reconstruction from human keratinocytes transduced with NT shRNA or TMEM45A shRNA. Scale bars: 50 μm. **(B)** Immunofluorescence detection of claudin-1 (Cldn-1) in ear epidermis from WT and KO mice. Scale bars: 25 μm.

Accordingly, the localization within the different epidermis layers of corneodesmosin (CDSN) and occludin (OCL), two actors of the epidermal barrier, was not altered in TMEM45A-deficient RHE (Fig 8A). Claudin-1 (Cldn-1) distribution in *Tmem45a*^*-/-*^ mouse epidermis was also unaffected (Fig 8B).

**Fig 9:**
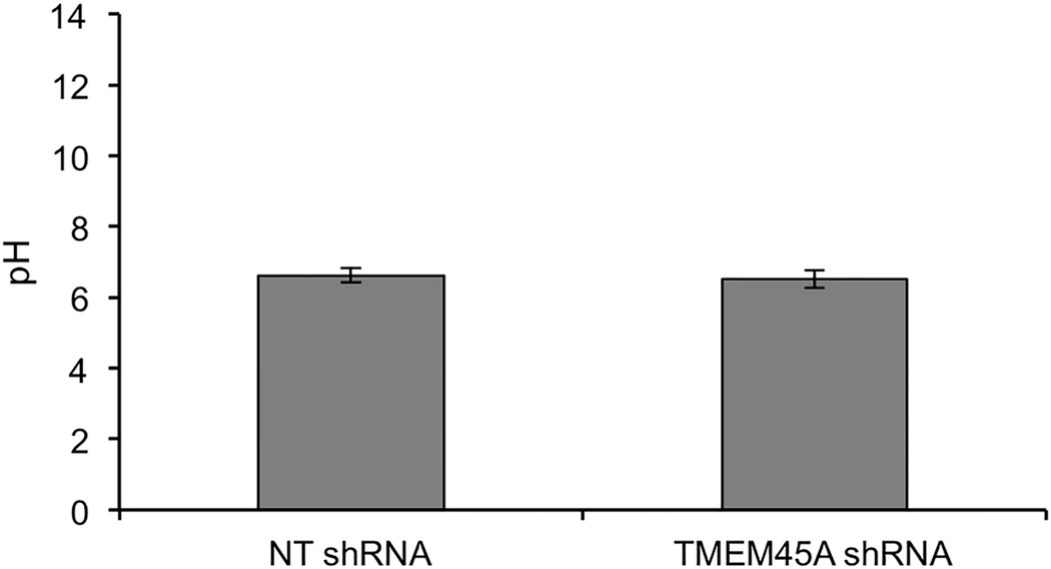
Unchanged pH at the surface of RHE after *TMEM45A* silencing. pH was measured at the surface of RHE obtained after 11 days from keratinocytes transduced with NT or TMEM45A shRNA (three different tissues prepared simultaneously were analyzed for each condition).

Finally, since the transmembrane location of TMEM45A makes it a possible proton-transporter, eventually contributing to acidification of the cornified layer, the pH value at the top of RHE was compared between tissues expressing or not TMEM45A. Again, we observed no difference (Fig 9).

In conclusion, invalidation of TMEM45A does not alter epidermal barrier formation and function, both in mice and in humans.

## Discussion

Because a strong correlation between TMEM45A expression and keratinization was previously observed, we hypothesized that this protein could be involved in this process [4]. We tested this hypothesis using human and mouse loss-of-function models: monolayer cultures of human keratinocytes, RHE and knockout mouse. Our observations clearly show that TMEM45A is dispensable for normal keratinocyte differentiation, epidermal development and maintenance. However, we cannot conclude that TMEM45A is not implicated in these processes. Indeed, mice knockout for different proteins, were shown to display functional alterations only under challenging conditions, or when other genes/proteins were simultaneously invalidated, pointing out to the phenomenon of biological robustness. In the case of the epidermis, challenging conditions could be exposure to UV-B or chemicals, injury or microbial infection. Epidermal response to such stresses in absence of TMEM45A will be analyzed in future experiments.

Of interest, TMEM45A has already been recognized as highly expressed in skin in other studies. For instance, its expression was ranked as 32^nd^ among the 100 skin-associated most expressed genes identified in silico, an observation confirmed by RT-qPCR analysis, as well as by immune detection of the encoded protein [6]. Despite its high expression in skin, mostly in keratinocytes and to a lesser extent in fibroblasts and endothelial cells, TMEM45A has been yet presented as a gene with unknown cutaneous function [6]. Whereas the expression of TMEM45A is poorly affected by inflammatory cytokines, it is upregulated in two pathological situations: psoriasis and actinic keratosis, both characterized by an abnormal and incomplete keratinization [6]. As TMEM45A is expressed in these poorly differentiated pathological keratinocytes, it must fulfill a function still present in these abnormal keratinocytes.

The consequences of *TMEM45A* deficiency could be masked by (over)expression of other genes, such as its paralog *TMEM45B*. This was demonstrated in many other cases of experimental gene invalidation including in *loricrin* knockout mice that show elevated levels of small prolin-rich proteins that constitute alternative precursors of the cornified envelope [21]. Such compensatory proteins remain to be identified in the case of *TMEM45A*.

The localization of TMEM45A in the trans-golgi/TGN of granular keratinocytes suggests a possible role for this protein. The two well-known functions of the trans-golgi/TGN are the glycosylation/sulfation of proteins, and the sorting of cargoes for correct trafficking. TMEM45A could be involved in enzymatic processes, including the maturation of lipids specific of the granular keratinocytes. In challenging conditions, TMEM45A could be implicated in the induced secretion of pro-inflammatory cytokines such as TNF-α or in the release of vesicular ATP [22].

In conclusion, although the previously reported strong correlation between TMEM45A expression and keratinization, the mouse and human loss of function models used in this study did not unveil a function for TMEM45A in epidermal morphogenesis, keratinization or barrier formation. The role of TMEM45A in epidermal granular layer remains thus to be elucidated.

## Supporting Information

S1 Fig Immunodetection of *TMEM45A* in MEFs. MEFs have been incubated 16 hours under normoxia or hypoxia (1% O_2_), fixed and immunolabelled for TMEM45A (green). The nuclei were stained with Hoechst (blue). Scale bars: 50 μm.
(TIF)

S2 Fig High variability of toluidine blue permeability in E17.5 mouse embryos. Pictures illustrate wild type (WT), knockout (KO) and heterozygous (HET) mouse littermates. Scale bars: 50 μm.
(TIF)

S1 File Supplementary material and methods. (PDF)
