# Supplementary figures and images for: TMEM45A Is Dispensable for Epidermal Morphogenesis, Keratinization and Barrier Formation
Source: PLoS One. 2016 Jan 19;11(1):e0147069. doi: 10.1371/journal.pone.0147069 (PMC4718520; doi:10.1371/journal.pone.0147069)

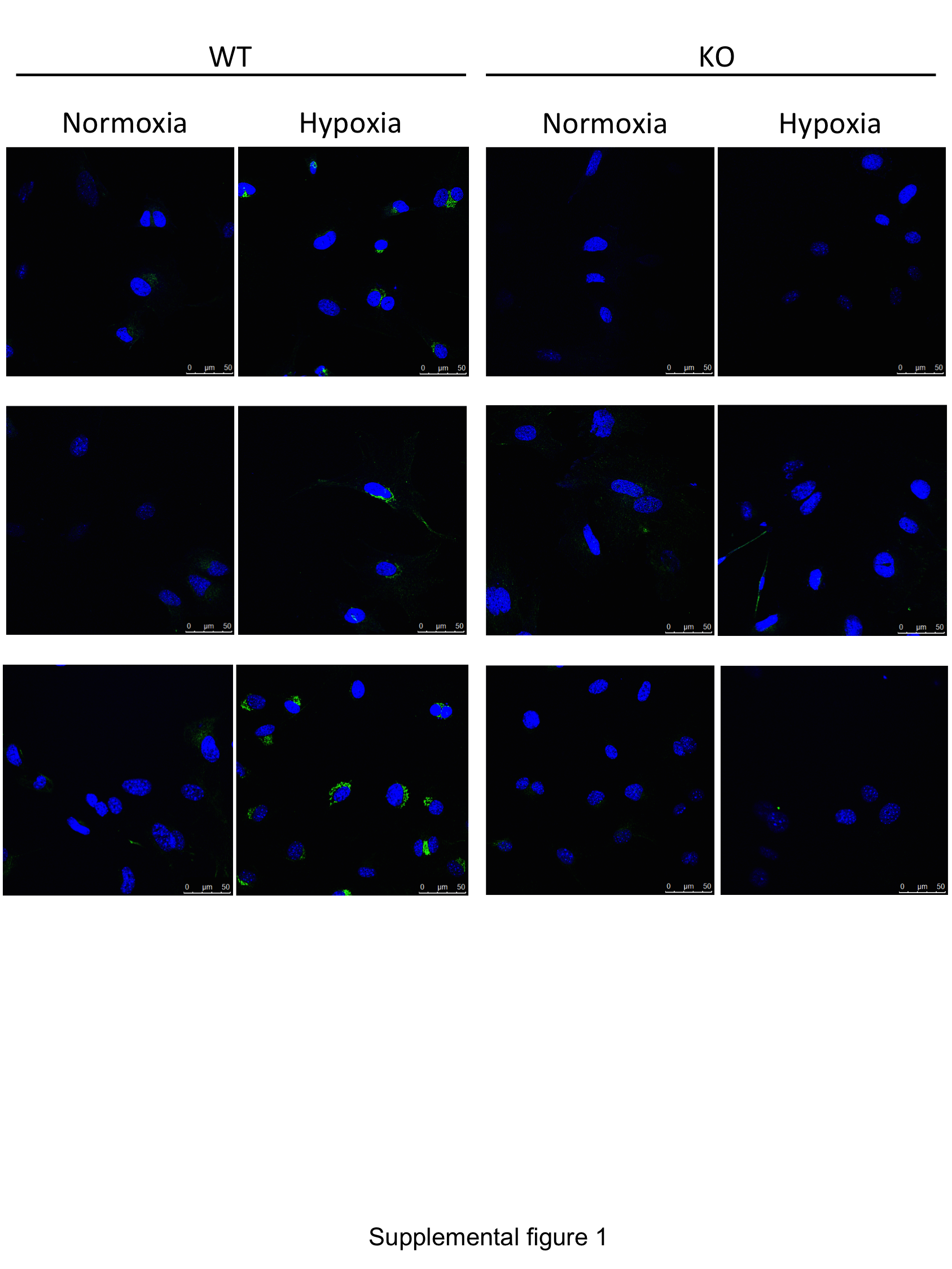

Supplement: S1 Fig — MEFs have been incubated 16 hours under normoxia or hypoxia (1% O2), fixed and immunolabelled for TMEM45A (green). The nuclei were stained with Hoechst (blue). Scale bars: 50 μm. (TIF) [file pone.0147069.s001.tif]

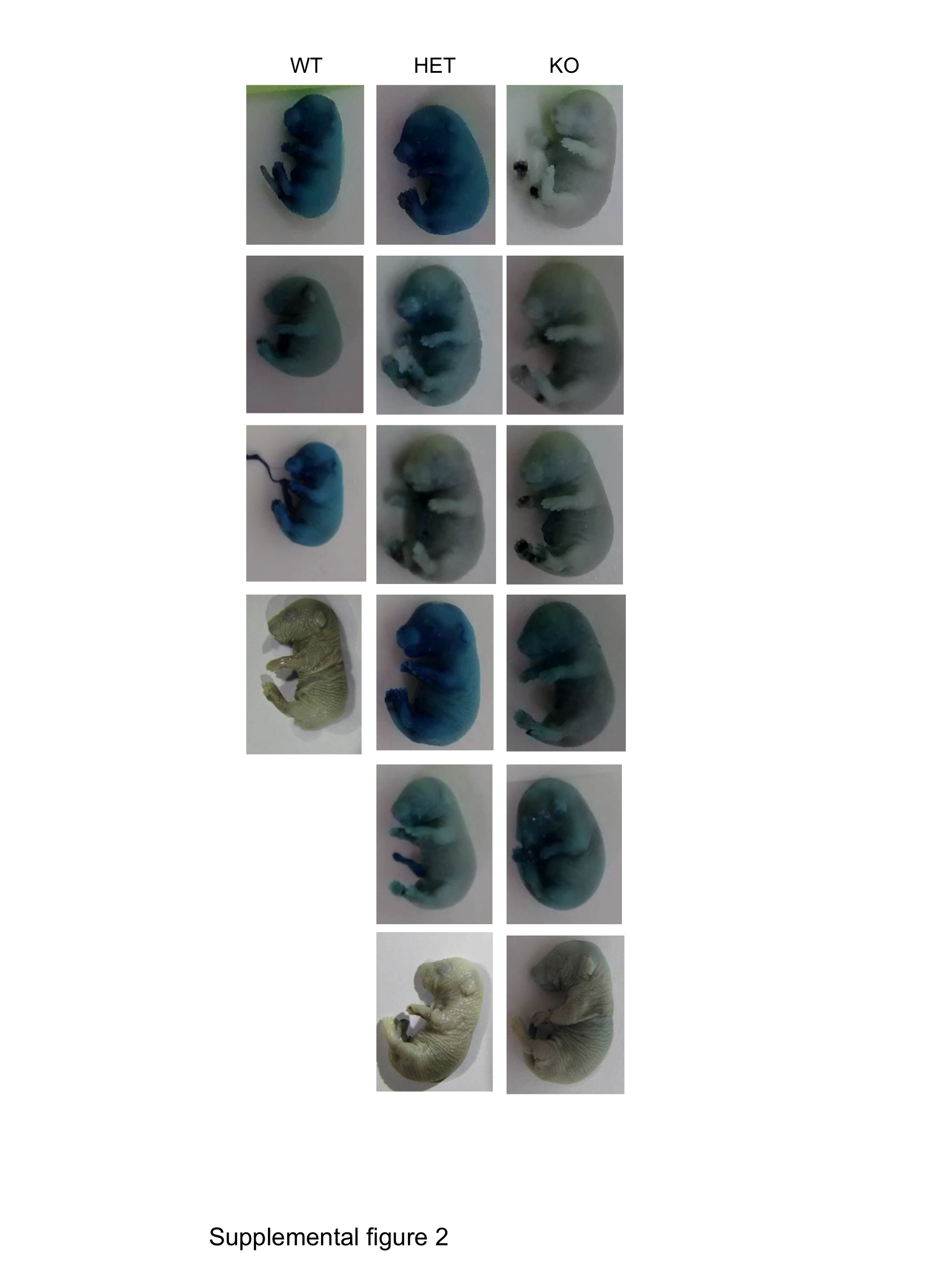

Supplement: S2 Fig — Pictures illustrate wild type (WT), knockout (KO) and heterozygous (HET) mouse littermates. Scale bars: 50 μm. (TIF) [file pone.0147069.s002.tif]
