# Supplementary material for: TMEM45A Is Dispensable for Epidermal Morphogenesis, Keratinization and Barrier Formation
Source: PLoS One. 2016 Jan 19;11(1):e0147069. doi: 10.1371/journal.pone.0147069 (PMC4718520; doi:10.1371/journal.pone.0147069)
Supplement: S1 File — (PDF) [file pone.0147069.s003.pdf]

Supplementary Material and Methods

Antibodies and chemicals

Rabbit anti-TMEM5A antibody #HPA024082 (dilution 1:100), mouse anti-IVL antibody #I9018 (dilution 1:200), rabbit anti-corneodesmosin antibody #HPA044730 (dilution 1:100), mouse anti-β-actin antibody #A5441 (dilution 1:10 000), protamine sulfate #P-4020 and Lucifer Yellow #L3510 were obtained from Sigma-Aldrich (Saint-Louis, MO). Hoechst 33258, TO-PRO-3 and Alexa fluor 488-conjugated goat anti-rabbit and anti-mouse antibodies (dilution 1:1,000), rabbit anti-claudin-1 #51-9000 antibody (dilution 1:50) and rabbit anti-occludin antibody #71-1500 (dilution 1:50) were purchased from Life Technologies (Carlsbad, CA). Mouse anti-KRT10 antibody #M7002 (dilution 1:100) was obtained from Dako (Glostrup, Denmark). Mouse anti-FLG antibody #MA1-91037 (dilution 1:50) and puromycin #BP2956-100 came from Thermo Fisher Scientific (Waltham, MA, USA). Mouse anti-KRT14 antibody #LL002 (dilution 1:50) was purchased from Santa Cruz (Dallas, TX). Rabbit anti-RPL13a #27655 (dilution 1:2,000) came from Cell Signaling (Danvers, MA).

Immunofluorescence detection in keratinocytes, reconstructed human epidermis and tissues

Reconstructed human epidermis and tissues were processed and embedded in paraffin, as described (1), or included in OCT compound to perform cryosections for TMEM45A detection. Paraffin-embedded sections were used for OCL, CDSN, KRT14, KRT10, IVL, CLD-1 and FLG detection. Paraffin-embedded sections were deparaffinized and rehydrated. For OCL, CDSN, CLD-1 and FLG detection, heat-induced antigen retrieval was performed by incubation in 10 mM citrate buffer pH 6 at 95°C for 20 minutes. Cryosections and cells on coverslips were fixed for 15 min in 4 % formaldehyde in phosphate-buffered saline (PBS) at pH 7.2. After rinsing in PBS, cryosections, paraffin sections and coverslips were incubated in

0.1 M glycine. Unmasked paraffin sections were blocked for 30 minutes in PBS containing 0.2% BSA. Cryosections, masked paraffin sections and coverslips were blocked and permeabilized in PBS containing 0.2% BSA and 0.02% Triton X-100 for 30 minutes. Incubations (for 45-120 minutes at room temperature, or overnight at 4°C) with primary antibodies diluted in PBS-BSA-Triton X-100 (cryosections, masked paraffin sections and cells on coverslips) or PBS-BSA (unmasked paraffin sections) were performed. Negative controls were generated by incubating sections and cells on coverslips in buffer without any primary antibody. After washings, sections and cells on coverslips were then incubated for one hour with secondary Alexa fluor antibodies. After washings, nuclei were finally stained with Hoechst 33258 or TO-PRO-3. Tissues and cells were analyzed under Leica TCS SP5II confocal microscope (Solms, Germany).

Primer sequences for human genes and for the puromycin resistance gene

Gene	Forward primer (5'-3')	Reverse primer (5'-3')
FLG	GGGCACTGAAAGGCAAAAAG	CACCATAATCATAATCTGCAC TACCA
IVL	TGAAACAGCCAACTCCAC	TTCCTCTTGCTTTGATGGG
KRT10	AATCA GATTCTCAACCTAACAAC	CTCATCCAGCA CCCTACG
KRT14	CGATGGCAAGGTGGTGTC	GGGTGAAGCAGGGTCCAG
LC1B	TGAGCCTAGAAGAACACAA	GAAGGAGAAGGAAGACGG
LOR	TCATGATGCTACCCGAGGTTTG	CAGACCTAGATGCAGCCGGA GA
Puromycin resistance gene	CGCCACATCGAGCGGGTCAC	GGCCGATCTCGGCGAACACC
RPLP0	ATCAACGGGTACAAACGAGTC	CAGATGGATCAGCCAAGAAG G
SPINK5	GACAATCTAAGAGTACAGCTTC CTT	TGTTGCCATGCATTTTTCCATC TG
TGM1	GTCGTCTTCCGGCTCGAA	TCACTGTTTCATTGCCTCCAAT
TPB	TCAAACCCAGAATTGTTCTCCTT AT	CCTGAATCCCTTTAGAATAGG GTAGA
TMEM45A	TTATGCAGTAACCATTGTCATCG TT	TGATTCTTGTTCTCGTTCAGCA TT

Primer sequences for murine genes

Gene	Forward primer (5'-3')	Reverse primer (5'-3')
Tbp	CACAAGGCCTTCCAGCCTTA	CACAGGAGCCAAGAGTGAAGA
Tmem45a	TTGCTGGCATTATGCATCAATC	CAGCCCTTCCTCAGCCTAGAC

Primers for *Tmem45a* have been designed so that the amplicon covers the exon 5 – exon 6 junction.

1. Frankart A, Malaisse J, De Vuyst E, Minner F, de Rouvroit CL, Poumay Y. Epidermal morphogenesis during progressive in vitro 3D reconstruction at the air-liquid interface. Experimental dermatology. 2012 Nov;21(11):871-5. PubMed PMID: 23163654.
